# Cross talk between the response regulators PhoB and TctD allows for the integration of diverse environmental signals in *Pseudomonas aeruginosa*

**DOI:** 10.1093/nar/gkv599

**Published:** 2015-06-15

**Authors:** Piotr Bielecki, Vanessa Jensen, Wiebke Schulze, Julia Gödeke, Janine Strehmel, Denitsa Eckweiler, Tanja Nicolai, Agata Bielecka, Thorsten Wille, Roman G. Gerlach, Susanne Häussler

**Affiliations:** 1 Department of Immunobiology, Yale University School of Medicine, New Haven, CT 06520, USA; 2 Institute of Molecular Bacteriology, TWINCORE GmbH, Centre for Clinical and Experimental Infection Research, a joint venture of the Hannover Medical School and the Helmholtz Centre for Infection Research, Feodor-Lynen-Strasse 7, 30625 Hannover, Germany; 3 Department of Molecular Bacteriology; Helmholtz Centre for Infection Research, Inhoffenstrasse 7, Braunschweig 38124, Germany; 4 Junior Research Group 3, Robert Koch-Institute, Wernigerode Branch, Burgstrasse 37, 38855 Wernigerode, Germany

## Abstract

Two-component systems (TCS) serve as stimulus-response coupling mechanisms to allow organisms to adapt to a variety of environmental conditions. The opportunistic pathogen *Pseudomonas aeruginosa* encodes for more than 100 TCS components. To avoid unwanted cross-talk, signaling cascades are very specific, with one sensor talking to its cognate response regulator (RR). However, cross-regulation may provide means to integrate different environmental stimuli into a harmonized output response. By applying a split luciferase complementation assay, we identified a functional interaction of two RRs of the OmpR/PhoB subfamily, namely PhoB and TctD in *P. aeruginosa*. Transcriptional profiling, ChIP-seq analysis and a global motif scan uncovered the regulons of the two RRs as well as a quadripartite binding motif in six promoter regions. Phosphate limitation resulted in PhoB-dependent expression of the downstream genes, whereas the presence of TctD counteracted this activation. Thus, the integration of two important environmental signals e.g. phosphate availability and the carbon source are achieved by a titration of the relative amounts of two phosphorylated RRs that inversely regulate a common subset of genes. In conclusion, our results on the PhoB and TctD mediated two-component signal transduction pathways exemplify how *P. aeruginosa* may exploit cross-regulation to adapt bacterial behavior to complex environments.

## INTRODUCTION

Two-component systems consist of a histidine (His) protein kinase that senses a signal input and a response regulator (RR) that mediates the output (1). They are ancient and evolutionarily conserved signaling mechanisms and represent the largest family of signaling systems in the bacterial kingdom. Two-component systems are involved in various signal transduction pathways and enable bacteria to sense, respond and adapt to a wide range of environments, stressors and growth conditions (2,3).

Most of the bacterial RRs are two-domain proteins consisting of a receiver module which is fused to a second output or effector activity domain (4). The output domain often is a DNA-binding module and functions as a transcription factor. Phosphorylation of the RR via its cognate histidine kinase (HK) thereby serves as a control element and e.g. dictates the ability of the RR to bind its target DNA sequence and to elicit a particular response. The great diversity within the sensing domains of the HKs and the effector domains of the RRs allow numerous variations within those two-component systems thus coupling diverse input stimuli to a wide range of output responses.

Bacterial two-component systems have been the subject of intense studies and many studies aimed at elucidating of how signal specificity in cells containing multiple two-component systems is achieved (5,6). Minimization of inappropriate cross-talk of the two-component systems is expected to allow the cell to carry out many diverse reactions robustly and provide a critical level of biological organization within the global transcriptional network. However, given the highly similar sequences and structures of the HKs and the RR proteins, there seems to be a great potential for cross-talk. Despite a variety of mechanisms to minimize unwanted, detrimental communication between distinct pathways, cross-regulation between signaling systems gives the opportunity to integrate information from multiple sources. This should be reflected by the pattern of connections among the two-component systems to build up higher-level-functions. Cross-regulation will allow distinct two-component systems to elicit some of the same responses via their respective RRs that regulate overlapping sets of genes (7). Cross-regulation can be archived at the level of phosphorylation where one RR is phosphorylated by various HKs or low molecular weight phospho-donors (8–10). However, there are also examples of two-component systems that cross-regulate one another at levels other than cross-phosphorylation (11). For example, it has been suggested that protein-protein interactions among the OmpR/PhoB RR subfamily members from *Escherichia coli* display significant interaction specificity suggesting potential cross regulation via RR hetero-pair interactions (12). Furthermore, RRs that are phosphorylated by distinct histidine kinases may confer to cross-regulation by binding to respective binding motifs in the promoter regions of a common sub-set of genes. Thus, those genes are regulated by more than on two-component system that work independently.

In this study, we used the split luciferase complementation assay as a versatile approach to systematically analyze functional protein–protein interactions of the RR PhoB with other members of the OmpR/PhoB RR subfamily in *Pseudomonas aeruginosa in vivo*. PhoB is activated under low phosphate medium conditions and the availability of phosphate has been recognized as an important nutrient and environmental signal that regulates virulence not only in *P. aeruginosa* (13,14) but also in various other bacterial species (15–19). We fused the split C- and N-terminal half of the secreted luciferase from the marine copepod *Gaussia princeps* (GLuc) to PhoB and to 16 RRs of the OmpR/PhoB subfamily, respectively and measured luminescence as a read out. Our results indicate that the protein-fragment complementation assay (PCA) can be used to measure OmpR/PhoB subfamily member interactions in *P. aeruginosa* and we identified a functional interaction of PhoB with the RR TctD *in vivo*. Further transcriptional profiling and chromatin immunoprecipitation (ChIP-seq) experiments uncovered the regulons and binding motifs of PhoB and TctD, respectively which were located close to each other in the promoter regions of an overlapping set of inversely regulated genes.

## MATERIALS AND METHODS

### Strains and growth conditions

Bacterial strains and plasmids used in this study are listed in Supplementary Table S1. *P. aeruginosa* strains (PA14 and PAO1) were cultivated in lysogeny broth (LB) at 37°C with shaking at 180 rpm. Alternatively, strains were cultivated under conditions, which either favored or inhibited *phoB* or *tctD* expression. Therefore strains were grown in DeMoss medium (10 g/l DL- Alanin; 20 ml/l Glycerol; 20 mM MgCl2; 0.1 M Na2SO4, 50 μM Fe(III)Citrat, pH 7.5) supplemented with low phosphate (0.8 mM K2HPO4) and high phosphate (4 mM K2HPO4), respectively, or M9 minimal medium with 10 mM citrate or 10 mM glucose. *E. coli* DH5*α* was routinely used for subcloning and propagation, *E. coli* S17-1 and WM3064 for bacterial conjugations. For plasmid selection and maintenance, antibiotics were added at the following final concentrations (μg/ml): for *E. coli*, ampicillin 100; gentamicin 15; for *P. aeruginosa*, carbenicillin 400; gentamicin 30. For gene expression L-arabinose (Sigma) was added to the culture medium in the induction range between 0.1% and 0.2% of L-arabinose.

### Plasmid and strain construction

Primers used are listed in Supplementary Table S2. The *gluc* gene encoding the luciferase enzyme of *Gaussia princeps* was amplified by polymerase chain reaction (PCR) from plasmid pBAD24-*gluc* (20) using a forward primer harboring a ribosomal binding site and the ATG start codon and a reverse primer with the stop codon TGA. PCR products were introduced into pHERD20T (21) under control of *P*_BAD_ resulting in pHERD20T-*P*_BAD_-*gluc*.

We constructed the pHERD20TGLucNNfus vector to perform split gaussia complementation assays. This vector harbored synthetic DNA fragments designed as follows: a cloning site for the gene of interest with EcoRI/SacI restriction sides in front of a linker sequence and the N-terminal part of *gluc* gene followed by stop codon and a KpnI restriction side; and a cloning site for the interacting gene of interest with KpnI/XbaI restriction sides in front of the linker sequence and the C-terminal part of the *gluc* gene followed by stop codon and HindIII side.

To construct pHERD20T-P_BAD_-RR-N*gluc-phoB*-C*gluc* the *phoB* gene was introduced within the KpnI and XbaI restriction site of pHERD20TGLucNNfus. To investigate the interaction of various response regulators with PhoB the respective genes (*bfmR, creB, gltR, amgR, pmrA, kdpE, parR, copR, pfeR, tctD*, PA1437, PA4381, PA2479, PA0929, PA3077, PA2523 and *pilH* as a negative and *phoB* as a positive control) were introduced within the EcoRI-SacI restriction site to generate a fusion protein with the NGLuc. In all genes introduced into the vector the respective stop codons were removed. Furthermore a ribosomal binding site was introduced (Supplementary Table S1).

A PAO1Δ*phoB* mutant was constructed according to a modified protocol using overlap extension PCR (22) and the gene replacement vector pEX18Ap. Regions up- and downstream of *phoB* were amplified by PCR. The primer *phoB*-up-RV and *phoB*-down-FW harbored complementary sequences (18 nt). The two corresponding PCR products were fused in a second PCR and the obtained fragment (the *phoB* gene harboring a deletion of 670 bp) was introduced in pEX18Ap resulting in pEX18Ap-up-*phoB*-down-*phoB*. The vector construct was transferred to PAO1 by two-parental mating using the donor strain *E. coli* S17-1. Selection of the PAO1Δ*phoB* deletion mutant was performed on sucrose (10%) and carbenicillin supplemented LB agar. The PAO1Δ*tctD* knockout mutant was generated by the gene replacement method with use of plasmid pEX18Ap and a gentamicin resistance cassette flanked by Flipase Recombination Target (FRT-Gm) originating from plasmid pPS856 (23). The mutant fragment was constructed by PCR extension overlap (24) during which the BamHI site was introduced between the upstream and downstream region of the target gene and this restriction site was used to insert the FRT-Gm. The resulting plasmid pEX18Ap*ΔtctD-Gm*, was transferred to PAO1 by two-parental mating using the donor strain *E. coli* S17-1. The occurrence of the double cross-over was checked by plating at least 30 colonies from the mating result on gentamicin and carbenicillin (400μg/ml) containing agar plates. Gentamicin resistant and carbenicillin sensitive bacteria were isolated and the insertion of the FRT-Gm cassette ensured by PCR. Finally the FRT-Gm cassette was removed from the chromosomal DNA with help of flipase encoded on pFLP3 plasmid (25). The PAO1Δ*phoB*Δ*tctD* double knock out mutant was generated by the use of the same protocol except that the pEX18Ap*ΔtctD*-Gm was transferred to the PAO1Δ*phoB* mutant instead of wild-type strain.

For purification of PhoB and TctD, the corresponding genes were amplified without stop-codons using primers listed in S1 and cloned into the NdeI–HindIII and NdeI–XhoI sites, respectively, of pET21a(+) thereby fusing the gene to a C-terminal His_6_-tag. Obtained constructs were confirmed by restriction analysis and sequencing prior to transformation into *E. coli* BL21(DE3).

For ChIP-seq experiments, pJN105-RBS-*phoB*-his8 was constructed using a *phoB* reverse primer additionally encoding eight copies of a His tag (8×His).

### Quantitative real-time PCR

Total RNA was extracted from cell pellets of 3 × 10^9^ bacteria using the RNeasy Plus Kit (Qiagen) in combination with Qiashredder columns (Qiagen) according to the manufacturer's instruction. The nucleic acids were treated with DNA-free™ kit from Ambion and the yield of total cellular RNA was determined by spectrophotometry (Nanodrop, Thermo Scientific). To quantify mRNA levels, the RNA was applied as a template for random-primed first-strand cDNA synthesis by using SuperScript III Reverse transcriptase (Invitrogen) according to the manufacturer's instructions. The cDNA was used as a template for quantitative PCR by using the SYBR Green Kit (Quiagen) and the Light Cycler®480 instrument (Roche). Primers are listed in Supplementary Table S2. The following cycling conditions were applied for all primer combinations in a reaction volume of 25 μl: 5 min at 95°C for denaturation, 40 cycles of amplification with 15 s at 95°C and 30 s at 60°C and final cooling step at 4°C. The cycle threshold (CT) was determined automatically by use of Real-Time 7300 PCR software. Expression was normalized against the ribosomal gene *rpsL* and the relative expressions were calculated from at least three biological and two technical replicas. The expression is given as the fold change with the standard deviations (26).

### Protein expression and purification

The purification of PhoB and TctD was performed as previously described (27). Briefly, *E. coli* BL21(DE3) cells carrying either pET21::phoB or pET21::tctD were grown in LB supplemented with 100 mg/ml ampicillin broth at 20°C. Expression was induced with 0.1 mM isopropyl-1-thio-b-Dgalactopyranoside (IPTG) at an OD600 of 0.5 to 0.7. After overnight incubation, bacteria were harvested and bacterial pellets were resuspended in cold lysis buffer (50 mM NaH_2_PO_4_, pH 8.0, 300 mM NaCl, 10 mM imidazole) containing 1 mM DTT, 1 mg/ml lysozyme, protease inhibitors (Complete mini, EDTA free, Roche) and Benzonase Nuclease (Novagen). Cells were then lysed by sonification and the cell-free supernatant was incubated with nickel-nitrilotriacetic acid agarose resin (Qiagen) for 1 h at 4°C. The resins were washed with lysis buffer and proteins were eluted with 50 mM NaH_2_PO_4_, pH 8.0, 300 mM NaCl and 250 mM imidazole. After SDS-PAGE analysis, fractions containing pure protein were pooled and dialyzed for 16 h at 4°C in 50 mM NaH_2_PO_4_, pH 8.0, 300 mM NaCl. The protein concentration was determined by using the Bradford-based Roti^®^-Quant solution following manufacture's instructions (Roth).

### Electrophoretic mobility shift assay (EMSA)

Electrophoretic mobility shift assays (EMSAs) were conducted using purified His_6_-PhoB and His_6_-TctD and fluorescence-labeled DNA fragments. The DNA probes of about 200 bp in length were generated by PCR amplification using primers listed in S2, which were ordered from Metabion (Martinsried, Germany) and carried the IRDye700 label (excitation wavelength λex 685 nm, emission wavelength λem 705 nm) at their 5′ end. As non-specific control a DNA fragment of the coding region of the house-keeping gene *gltA* was used. For binding reaction, 20 ng of specific DNA probe was incubated with varying amounts of purified protein in binding buffer (20 mM Tris–HCl, pH 7.5, 50 mM NaCl, 1 mM EDTA, 1 mM DTT, 100 μg/ml BSA, 150 ng/μl poly(dIdC), 5% glycerol; (28). The reaction samples (15 μl) were incubated at room temperature in the dark for 30 min before being electrophoresed on native 5% Mini-Protean^®^ Tris-borate-EDTA polyacrylamide (TBE) precast gels (Bio-Rad) in TBE buffer (89 mM Tris base, 89 mM boric acid, 2 mM EDTA) at 4°C at 90 V for 1.5 h. Gels were scanned at 0.5 μm resolution with a Odyssey CLx Infrared imaging system (LI-COR) using the BPFR700 filter and 689 nm as excitation wavelength. Images were conducted with Image Studio® software. Each experiment was performed at least in triplicates.

### Recombinant *Gaussia* luciferase assays

Bacterial cell lysates were cleared by centrifugation and stored at −20°C (storage of even several days did not lead to significant losses of Gluc activity). To measure Gluc activity 60 μl of Coelentrazin (10 μM, dissolved in phosphate buffered saline) (Biotium, Hayward, CA, USA) was subjected to 100 μl of lysate within a 96-well polystyrene plate. Luminescence measurements were carried out in 96-well white flat-bottom plates by the use of a Centro XS3 LB 960 microplate Luminometer (Berthold Technologies). The reaction was started by well-wise addition of the substrate, wells were shaken for 1 second and luminescence was measured after 4 s for 4 s at 480 nm. Luminescence is given as the relative luminescence of 100 μl of the cultures measured in a 96-well plate divided by the OD_600_ (relative luminescence units, RLU/OD_600_). All results represent the mean of at least two independent replicates.

### mRNA profiling

For mRNA profiling, two independent experiments were performed and each experiment included pooling of three individual main cultures. RNA was prepared from PA14 wild-type, PA14 *tctD::Tn* and PA14 ΔPhoB strains grown in DeMoss medium under low phosphate concentrations. RNA extraction, cDNA library preparation and deep sequencing were performed as previously described (29). In brief, cells were harvested after addition of RNA protect buffer (Qiagen) and RNA was isolated from cell pellets using the RNeasy plus kit (Qiagen). mRNA was enriched (MICROBExpress kit (Ambion)), fragmented and ligated to specific RNA-adapters containing a hexameric barcode sequence for multiplexing. The RNA-libraries were reverse transcribed and amplified resulting in cDNA libraries ready for sequencing. All samples were sequenced on an Illumina Genome Analyzer II-x in the Single End mode with 66 cycles.

### Quantification of gene expression

Sequence reads were separated according to their barcodes and barcode sequences were removed. The reads were mapped to the genome sequence of the reference strain wild-type *P. aeruginosa* PA14 using Stampy (30) with default settings. The R package DESeq (31) was used for differential gene expression analysis. Genes were identified as differentially expressed if they fulfilled the following criteria: (i) their logarithmic fold change, log_2_FC, was higher than 1 or lower than −1 in a comparison of the *phoB* or *tctD* mutant with the wild-type strain and (ii) the Benjamini–Hochberg corrected *P*-value, *P*_adj_, was smaller than 5%.

### Chromatin immunoprecipitation followed by deep sequencing

For detection of PhoB-bound promoters one ChIP-seq experiment was performed for the PhoB overexpressing strain and one for the wild-type control, and each experiment included pooling of two individual cultures of 50 ml. ChIP-seq was applied to four 20-ml cultures of PA14 (pJN105-RBS-sigXhis8) and PA14 (pJN105) as a control strain under the same culture conditions as described in the section on mRNA profiling. Detailed description of the experimental protocols is available in (32). The DNA libraries were sequenced on an Illumina Genome Analyzer IIx in the single end mode with 72 cycles.

### Analysis of ChIP-seq data

Adapter sequences were removed using the fastq-mcf script that is part of the EA-utils package (33). We used the Bowtie aligner (34) to map the reads against the PA14 reference sequence. Model-based analysis of ChIP-seq (MACS) (35) was applied for peak detection using a *P*-value cutoff of 5% and shift size 30 for the peak modeling, using the control sample for false discovery rate estimation. Promoter hits were considered significant when they had an enrichment factor (EF) of at least 32 and a *P*-value of 0.0001.

### Definition and functional profiling of the PhoB regulon

Motif search was performed using the MEME suite (36) on promoter regions whose respective genes (i) showed a PhoB-dependent downregulation in the PA14 ΔPhoB and (ii) were identified in the ChIP-seq experiment. These criteria were met by 40 candidates, whereas there was a significant overlap between the genes detected by ChIP-seq and RNA-seq approaches (hypergeometric test, *P*-value of 10^−14^). Promoter regions were defined as sequences 500 bp upstream of the respective start codon. The MEME parameters ‘-anr –minw 15 –maxw 30’ were specified and the DNA option ‘search given strand only’ was activated. Furthermore, a PA14 background Markov model of first order was supplied. Next, the obtained motif was submitted to MAST (36) to identify putative PhoB binding sites in all promoter regions of the *P. aeruginosa* PA14 genome. Strand-specific promoter hits with a *P*-value cutoff of 10^−3^ were regarded as significant, summing up to 720 candidate promoters. To define the primary PhoB regulon, genes were selected which fulfilled at least two of the following three criteria: (i) exhibited PhoB-dependent regulation of expression, (ii) had a promoter that was enriched in ChIP-seq experiments and (iii) had a promoter that contained a PhoB binding site. Finally, statistical significance of the candidate genes was checked by performing a hypergeometric test on the intersections ChIP-seq/RNA-seq, RNA-seq/Motif search and ChIP-seq/Motif search, where all comparisons but RNA-seq/Motif search returned *P*-values < 0.05. The final set of 188 genes was functionally characterized using the PseudoCAP annotation (30) following the steps described in (32). An enrichment value of 1.5 was defined as over-representation and a value of 0.66 as under-representation.

## RESULTS

### Recombinant *Gaussia* luciferase activity in *P. aeruginosa*

**Figure 1.**
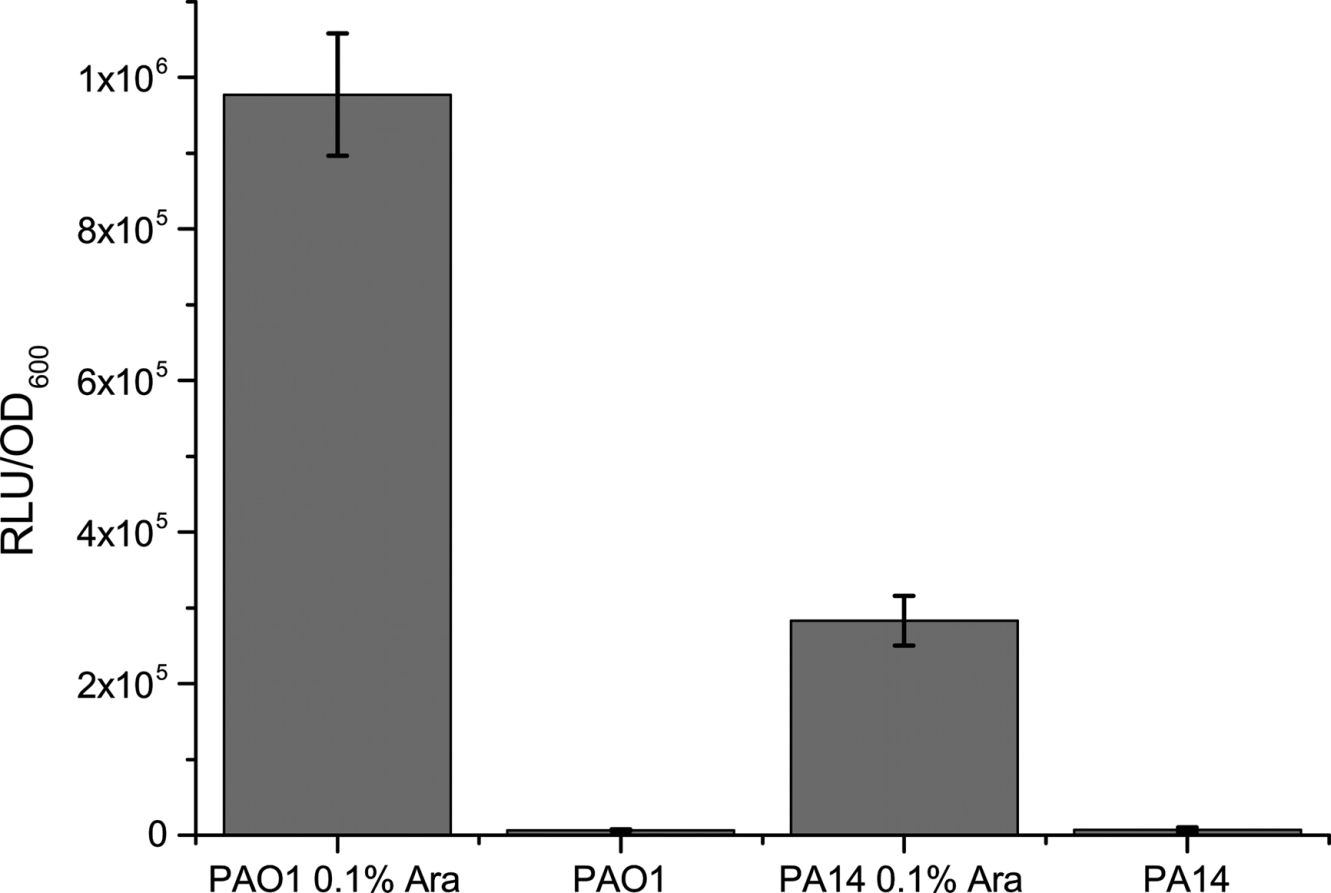
The luminescence signal expressed as Relative Luminescence Units normalized to the bacterial OD_600_, RLU/OD_600_, was recorded after bacterial growth to late exponential growth phase (7 h, OD_600_ = 2.0) under the addition or absence of 0.1% L-Arabinose. Mean values and standard deviation of three biological replicas are shown.

In this study we used the small (18kDa), secreted luciferase from the marine copepod *Gaussia princeps* (GLuc) as a sensitive marker and reporter system. We first cloned the codon-optimized gene encoding GLuc into the shuttle vector pHERD20T, which induces protein expression under the control of a *P*_BAD_ arabinose inducible promoter and expressed this vector (pHERD20T-GLuc) in *P. aeruginosa* PAO1 as well as PA14. The highest expression of the GLuc was observed in the induction range between 0.1% and 0.2% of L-Arabinose for both *P. aeruginosa* strains. Figure 1 demonstrates bioluminescence activity of transformed PAO1 and PA14 *P. aeruginosa* strains as measured during the late exponential growth phase. Of note, luminescence activity of living bacterial cells after addition of the substrate was much higher in PAO1 as compared to PA14. This variation seems to be due to a difference in the activity of efflux pumps or the permeability of the cell membrane for the GLuc substrate coelenterazine, since testing of cell lysates of both strains did not show a differential in luminescence (data not shown). Monitoring luminescence throughout the growth phase of *P. aeruginosa* PAO1 cultures (Supplementary Figure S1) revealed that luminescence was not detected before 5 h of growth. The highest luminescence intensities were observed in the second stage of exponential growth and dropped to less than half maximum levels when bacterial cells entered the stationary phase.

### Split *Gaussia* protein fragment complementation assay to monitor PhoB protein functional interactions with other response regulators of the OmpR/PhoB subfamily in *P. aeruginosa*

**Figure 2.**
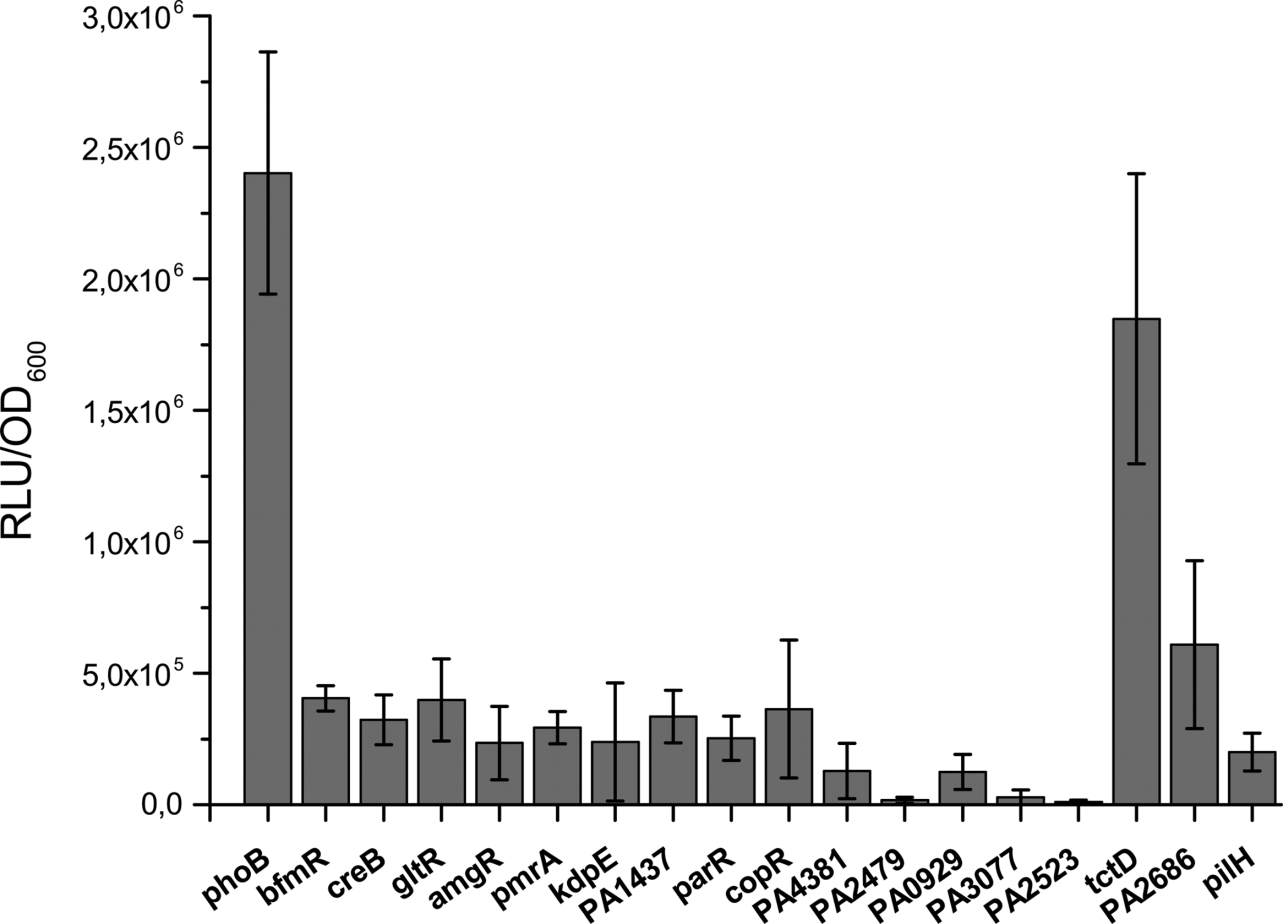
The *Pseudomonas aeruginosa* PAO1 *phoB* deletion mutant carrying split GLuc constructs was grown in DeMoss minimal medium under low phosphate conditions (0.8 mM P_i_) until exponential phase. All samples were induced with 0.01% Arabinose. Relative luminescence values of split N-terminal half of the luciferase to the response regulator PhoB and C-terminal luciferase to 16 response regulators of the PhoB subfamily in comparison to the Phob/PhoB split Gaussia samples are depicted.

PhoB acts as a homodimer to induce the transcriptional expression of the PhoB/R-dependent (PHO) regulon. Recently, Gao *et* *al*. have systematically analyzed possible protein–protein interactions among 14 OmpR/PhoB subfamily members from *E. coli* and found that RRs of the OmpR/PhoB subfamily displayed significant interaction specificity (12). However, they also found-albeit weak-hetero-pair interactions, suggesting potential cross regulation between distinct two-component pathways. In this study we evaluated the use of the split *Gaussia* luciferase PCA as an *in vivo* assay to screen for functional interactions between PhoB subfamily RR members in the bacterial model organism *P. aeruginosa*. We therefore fused the split C-terminal half of the luciferase to the RR PhoB and the N-terminal half to 16 RRs of the Ompr/PhoB subfamily (BfmR, CreB, GltR, AmgR, PmrA, KdpE, ParR, CopR, PfeR, TctD, PA1437, PA4381, PA2479, PA0929, PA3077, PA2523 and to PilH as a negative and PhoB as a positive control), respectively and measured luminescence as a read out in the PAO1 *phoB* deletion mutant. The strongest signal was observed for the reporter strain where both split GLuc were fused to PhoB. Although none of the fusions with other RRs reached the bioluminescence levels of the PhoB homo-dimerization signal, the TctD/PhoB split constructs gave a clear luminescence signal (Figure 2). TctD is a RR of the two component system TctDE, which was described to regulate expression of the *tctCBA* operon encoding a tricarboxylate transport system in *Salmonella* Typhimurium (37). In *P. aeruginosa* the homologous *tctCBA* operon harbors an additional gene, *opdH*, encoding for an outer membrane protein (38).

### The response regulators PhoB and TctD have opposing effects on the transcription of a common set of genes

To determine whether there is a common set of genes regulated by both PhoB and TctD, we performed RNA-seq under low phosphate medium conditions. We therefore selected the PA14 *tctD*::Tn transposon mutant from the PA14 Harvard Medical School library (39) and generated a *phoB* deletion mutant in *P. aeruginosa* PA14. All differentially expressed genes in both mutants as compared to the PA14 wild-type are listed in Supplementary Table S3. A total of 96 genes were downregulated in the Δ*phoB* mutant as opposed to PA14, whereas 10 genes were upregulated at least two-fold. Interestingly, in the *tctD* transposon mutant only 14 genes (organized in 10 operon structures) were found to be differentially regulated. Among them was the *opdHtctCBA* operon. Strikingly, with the exception of this operon, *tctD* itself and one additional gene (*ugd*), all of the TctD regulated genes were also regulated by PhoB. However, genes that were upregulated in the *tctD* mutant were downregulated in the *phoB* mutant (Table 1).

**Table 1.** Differentially expressed genes in the PA14 *tctD*:Tn and their level of expression in the PA14 *phoB* deletion mutant

			Δ*phoB* vs PA14 WT		*tctD*:Tn vs PA14 WT		PhoB ChIP-Seq		**	**
Gene ID	PAO1 ID	name	log2FC	*P*-value	log2FC	*P*-value	log2FC	*P*-value	PhoB	TctD
PA14_04550	PA0347	***glpQ***	−2.03	5.56E-03	2.01	8.3E-05	8.40	2.23E-272	+	+
PA14_13330	PA3910		−4.55	7.17E-30	1.31	2.5E-03	5.70	1.13E-47	+	+
PA14_20491	PA3368		−1.91	6.08E-03	1.61	1.2E-02	6.05	9.89E-66	+	+
PA14_21610	PA3280	***oprO***	−5.06	1.67E-35	1.87	1.0E-07	7.08	2.55E-202	+	+
PA14_22600		***grtA***	−2.07	1.96E-03	1.68	1.7E-03	*8.47	*0.00	*	*
PA14_38360	PA2022	***ugd***	0.34	1	1.83	1.4E-02	8.55	0.00	+	+
PA14_55320	PA0696		−2.85	1.37E-06	1.67	4.1E-03			(+)	+
PA14_56560	PA4350		−3.01	3.99E-11	1.79	9.3E-06	8.40	0.00	+	+
PA14_56570	PA4351		<-10	5.67E-10	1.84	4.3E-04	*	*	*	*

The enrichment of promoter regions as determined by the use of PhoB ChIP-seq experiments and the presence of DNA-binding motifs in the promoter regions are also shown.

*detected in the promoter region of the operon.

**P-value cutoff 10^−3^.

(+)longer spacer or weak motif.

### Genome-wide mapping of PhoB-binding regions

**Figure 3.**
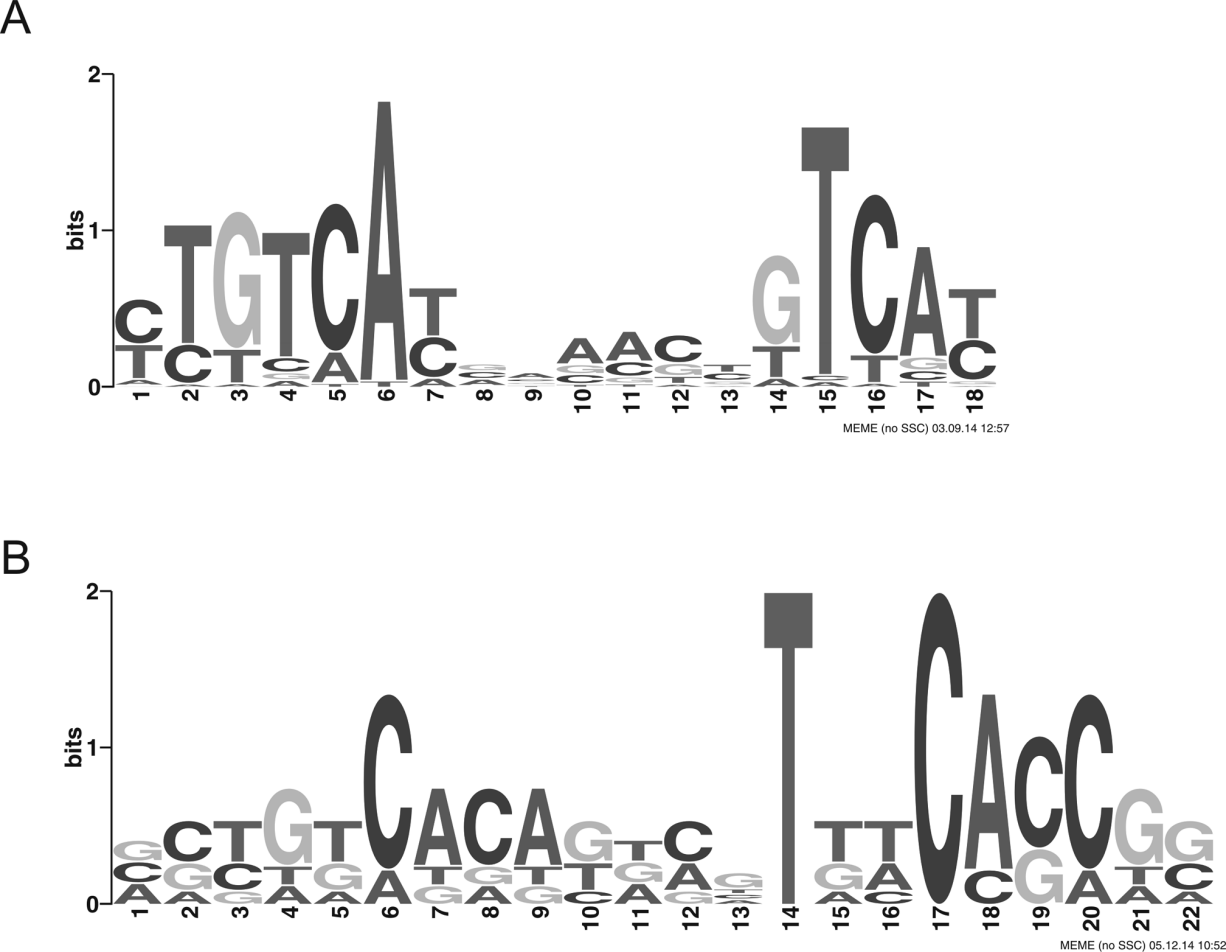
(**A**) PhoB binding consensus sequence recovered *de novo* in the promoters of genes showing PhoB-dependent regulation as confirmed by ChIP-seq and RNA-seq data. The motif is based on 30 out of the 40 submitted promoters. The *E*-value of the motif computed with MEME (36) is 2.9 × 10^−33^. (**B**) TctD binding consensus sequence recovered *de novo* in the promoters of nine genes showing TctD-dependent regulation (Supplementary Table S3).

We next complemented our transcriptome data with chromatin immunoprecipitation (ChIP-seq) experiments to define the primary regulon of *P. aeruginosa* PhoB and thus to differentiate direct from indirect PhoB-dependent regulation of genes. We constructed a variant of PhoB containing a 8xHis-tag, introduced this into the PA14 Δ*phoB* and sequenced PhoB bound genomic DNA upon L-arabinose induced *phoB* overexpression and growth under low phosphate conditions. All genomic regions showing significant (*P*-value ≤ 0.05) ChIP-seq enrichment are listed in Supplementary Table S4. As many as 276 of those genomic regions showed at least 32-fold enrichment in the ChIP-seq approach (log_2_FC ≥ 5, *P*-value ≤ 10^−^^4^). A total of 91.7% of these regions were located within 200 base pairs (bp) upstream of the putative translation start sites (ATG) of altogether 590 genes as some of the enriched regions could be assigned to two adjacent genes with opposite orientation. We next applied a motif search using the MEME suite (27) on those promoter regions whose respective genes exhibited a PhoB dependent regulation of expression (as listed in Supplementary Table S3) and were identified in the ChIP-seq experiments to bind PhoB. Figure 3A displays the sequence logo with the PhoB consensus sequence. A bipartite motif containing two (T)GTCAT boxes separated by a spacer of 5 nt can be recognized which is very similar to that described previously (13).

We also applied a motif search on those promoter regions whose respective genes exhibited a TctD dependent regulation of expression (overall eight promoter regions). The putative TctD binding motif is depicted in Figure 3B.

### Defining the primary PhoB regulon

**Figure 4.**
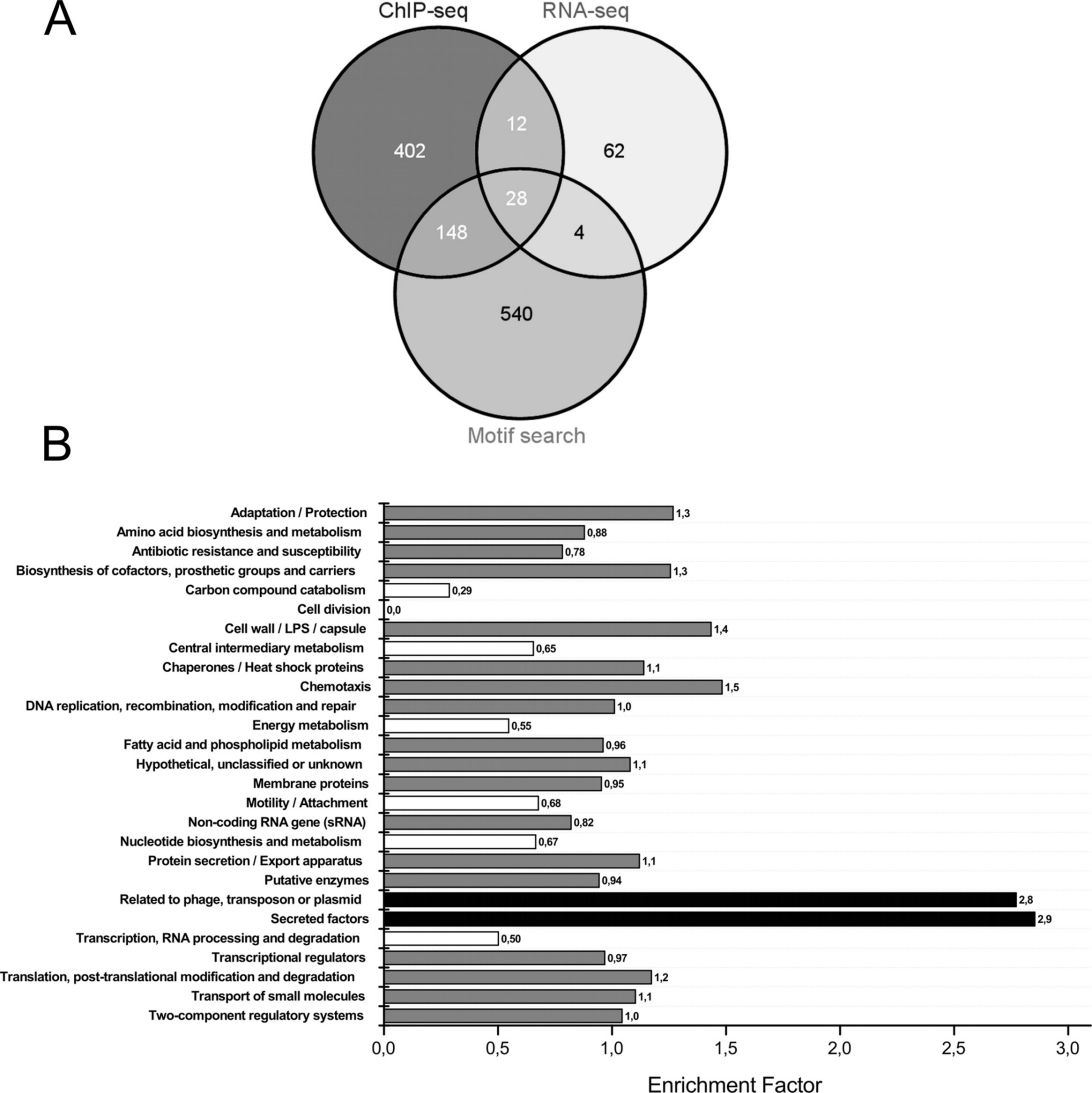
(**A**) Quantitative analysis of the PhoB regulon using ChIP-seq, RNA-seq and motif data. Genes in all intersections except RNA-seq/Motif search were defined as parts of the primary PhoB regulon. (**B**) Functional analysis of the PhoB regulon in terms of enrichment of Pseudocap classes. Significantly overrepresented classes (Enrichment values ≥ 1.5, *P*-value ≤ 0.05) are shown in black, while underrepresented classes (Enrichment values ≤ 0.66) are shown in white.

To further characterize the primary regulon of PhoB, we identified those genes that (i) exhibited a PhoB dependent regulation of expression (106 genes, as listed in Supplementary Table S3), (ii) were identified in the ChIP-seq experiments to bind PhoB in the promoter region (590 genes) and (iii) displayed a PhoB motif (Figure 3A) in their promoter region (720 genes) (41). A total of 188 genes fulfilled at least two of those three criteria (Figure 4A) and are listed in Supplementary Table S5. To functionally profile genes of the primary PhoB regulon, we used the PseudoCAP annotation (30). Two functional groups (‘Secreted factors’ and ‘related to phage, transposon or plasmid’) were strongly enriched in the group of PhoB dependent genes (Figure 4B). Interestingly, for all but one (PA0696) of the *tctD/phoB* co-regulated genes, a PhoB as well as a TctD binding motif (Figure 3, Table 1) was found in the promoter region. In contrast only a TctD binding motif was found in the promoter region of the *tctD* gene and the o*pdHtctCBA* operon. These results indicate that while TctD regulates expression of its encoding gene and the o*pdHtctCBA* operon, both, TctD as well as PhoB act as transcriptional regulators on a common subset of genes. However, rather than exhibiting a coherent activity the transcriptional regulators PhoB and TctD seem to act as opponents.

### Consensus PhoB/TctD binding box

**Figure 5.**
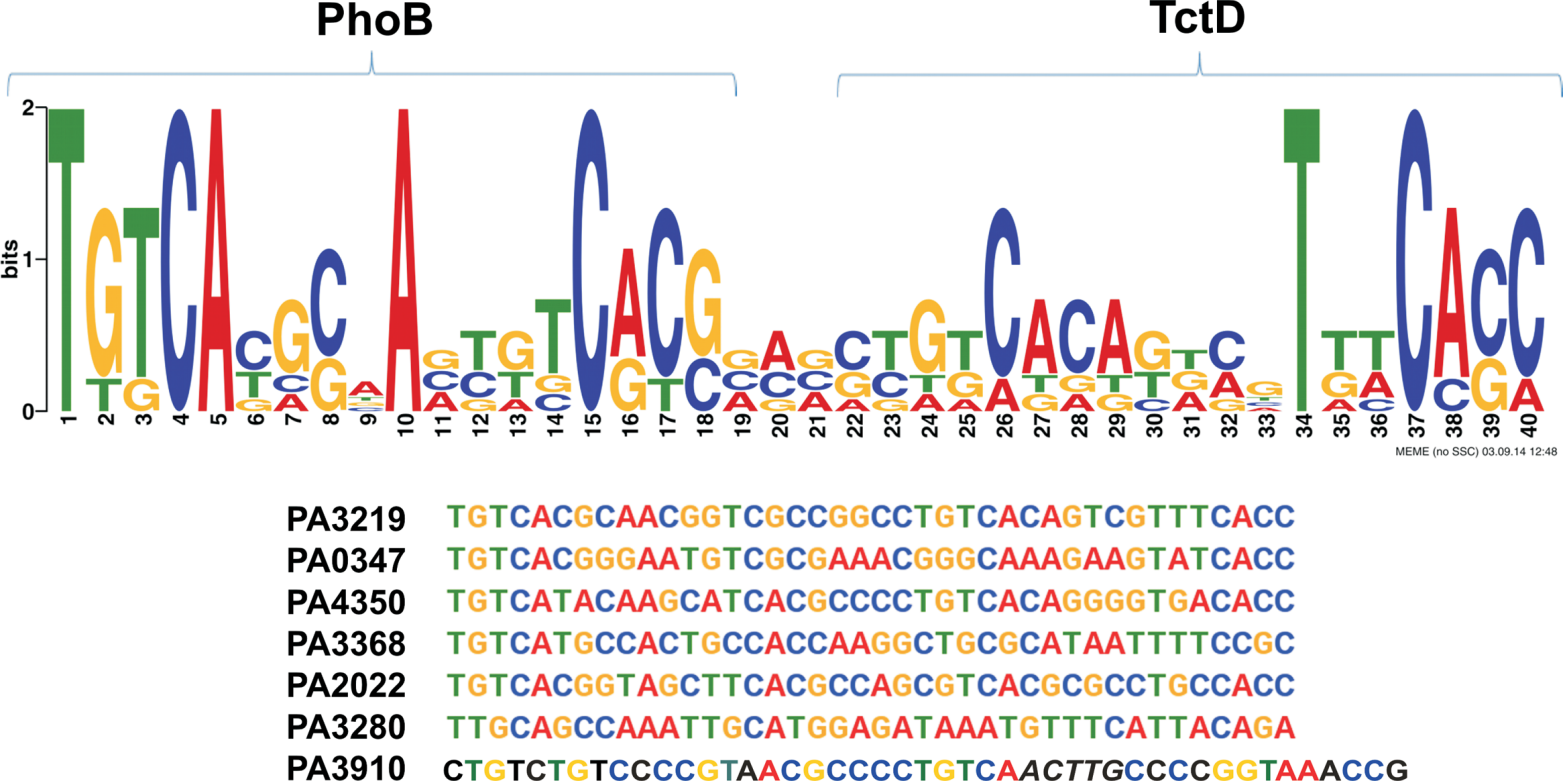
PhoB-TctD consensus binding box found *de novo* in the promoters of co-regulated genes. The aligned promoter sequences are ordered from top to bottom by increasing *P*-values as returned by MEME. Only the strongly conserved sequences found in the first 6 promoters were used to build the position weight matrix. The PhoB-TctD box present in the PA3910 promoter has a 5bp longer spacer (shown in italics) than the consensus motif. In the PA3910 sequence only the nucleotides present at least once at the corresponding positions in the consensus alignment are colored.

**Figure 6.**
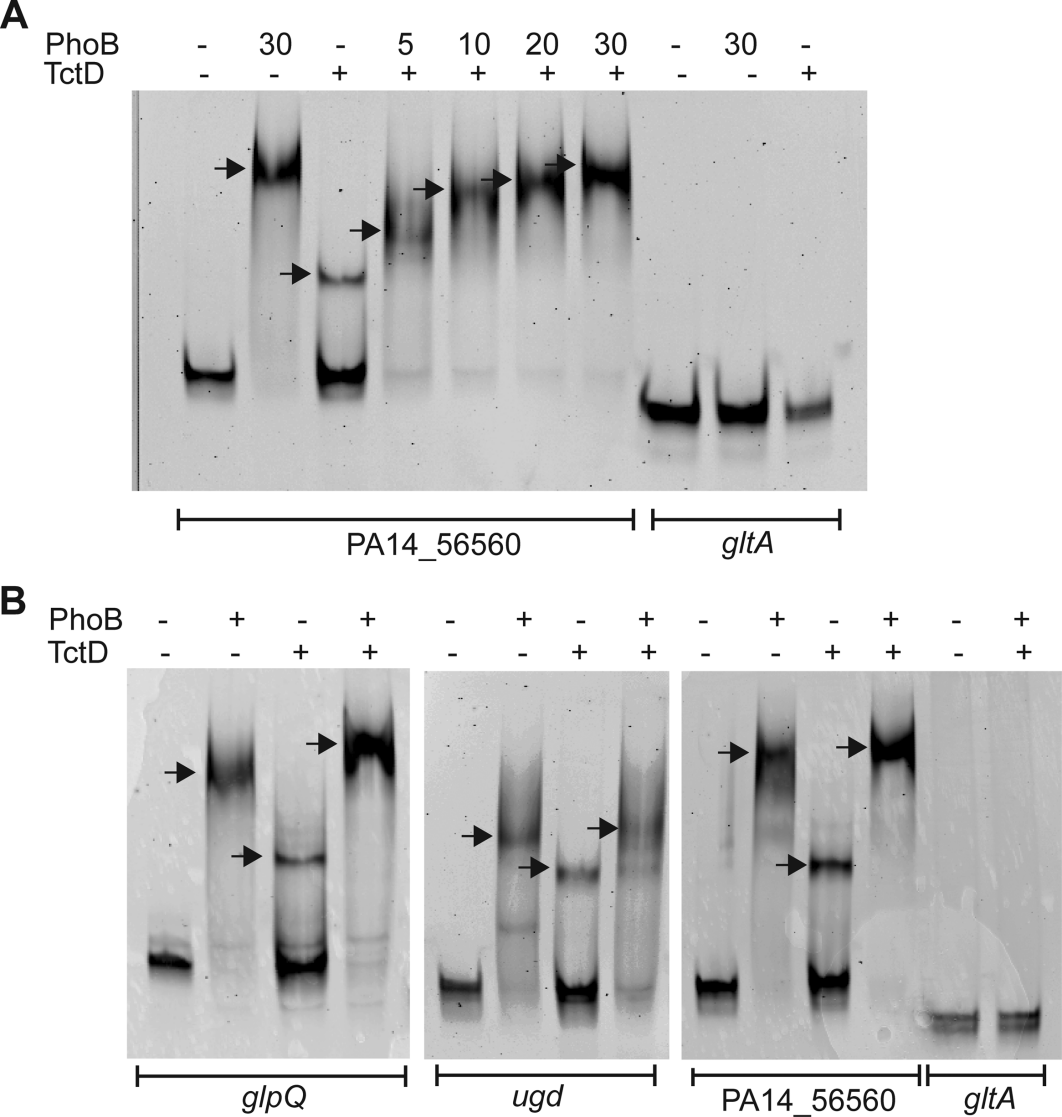
Electrophoretic Mobility Shift Assay (EMSA) of IRDye 700-labeld DNA probes in the presence of PhoB and TctD. The promoter regions of selected genes carrying putative PhoB- and TctD-binding motifs were amplified and incubated with purified PhoB and TctD, respectively. (**A**) Promoter region of PA14_56560 was incubated with 30 μM TctD and varying concentrations of PhoB given in μM. (**B**) Selected promoter probes were incubated with 30 μM PhoB (+) and 30 μM TctD (+). A DNA probe of *gltA* served as a non-specific control. Each experiment was performed at least in triplicates.

Since our split *Gaussia* protein fragment complementation assay indicated a functional protein-protein interaction of PhoB and TctD, we hypothesized that this interaction might be due to simultaneous binding of the two RRs to the promoter regions of the co-regulated genes. We therefore used the sequence of the seven promoter regions of the co-regulated genes as the basis for elucidating the *de novo* binding motif of a combined binding of PhoB and TctD. Remarkably, as depicted in Figure 5 we found for six of the seven promoter regions a consensus sequence, which comprises the PhoB box and if extended by additional 22 nt also the TctD binding motif. The sixth promoter region (PA3910)—albeit also containing both the PhoB and the TctD binding motif—exhibited a longer interspace region. These results suggest that PhoB and TctD bind as homo-dimers to the DNA at a close and defined distance to each other. To confirm DNA binding activity of the two RRs we performed mobility shift assays of suitable 200 bp DNA fragments containing in the 5′ flanking regions of three exemplarily chosen genes that harbored the PhoB/TctD binding motif (*glpQ*, PA14_56560 and *ugd*). Thirty micromolar of TctD (maximal concentration which could be applied) partially shifted the DNA, whereas already lower concentrations of PhoB to completely shift the DNA, indicating a stronger binding affinity of PhoB under the applied *in vitro* conditions. Under the same stringent conditions, both proteins failed to associate to a 200-bp DNA fragment of the coding region of the *gltA* gene, used as a negative control for binding specificity (Figure 6). Simultaneous addition of PhoB and TctD lead to more defined and slightly super-shifted bands, indicating a specific recognition of the promoter regions by PhoB and TctD.

### Functional profiling of PhoB and TctD activity

**Figure 7.**
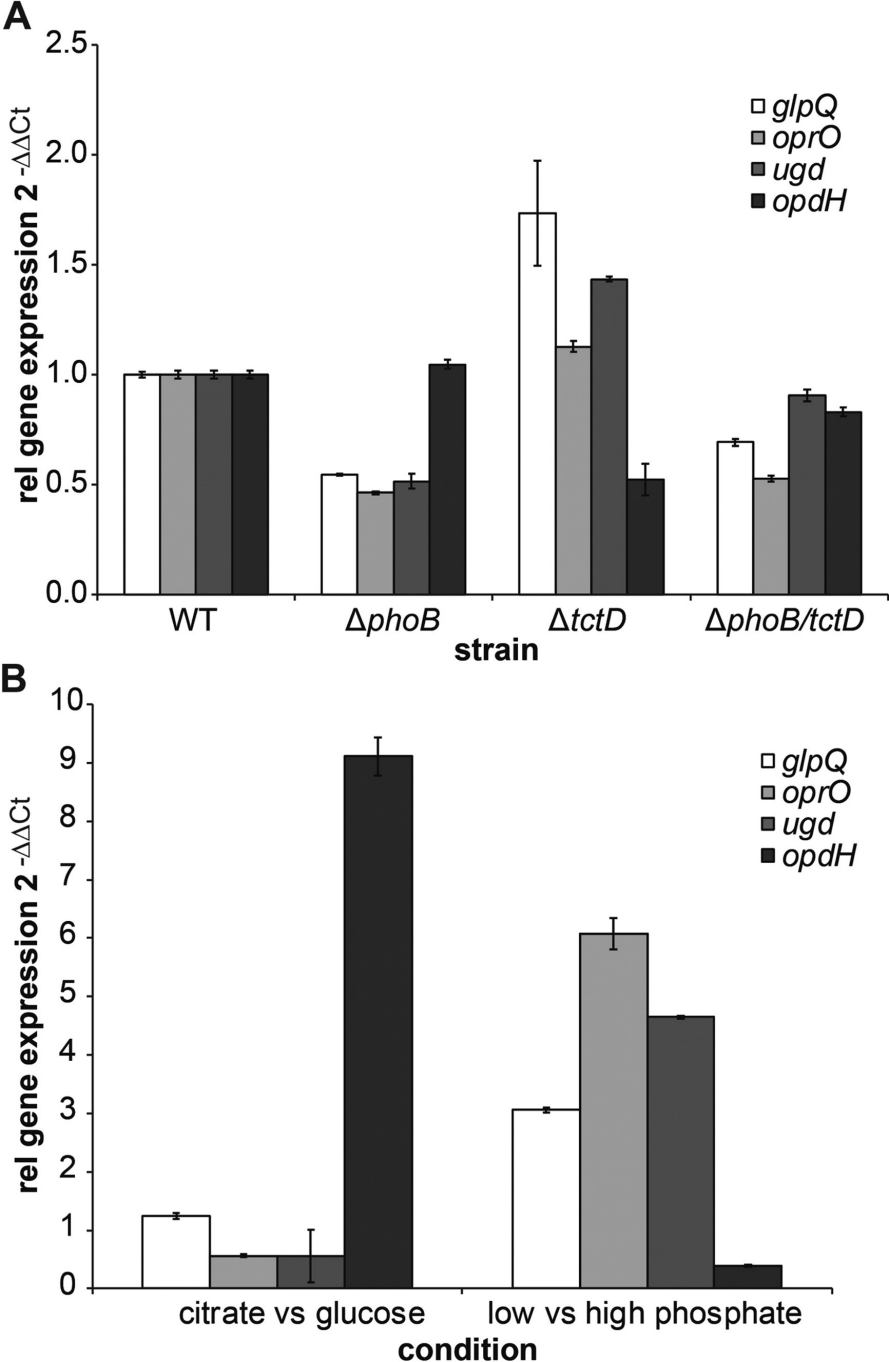
Quantitative analysis of gene expression of PhoB- and TctD-regulated targets. mRNA expression levels were determined by real-time RT-PCR assay using SYBR^®^-Green. The threshold cycle (Ct) values were used to quantify the PCR product, and the relative expression level of the target genes was expressed as 2^−ΔΔCt^. Expression was normalized against the ribosomal gene *rpsL* (ΔCt). (**A**) *Pseudomonas aeruginosa* PAO1 wild-type, Δ*phoB*, Δ*tctD* and Δ*phoB*Δ*tctD* cells were cultivated in LB medium and the expression profile of selected genes in mutants was normalized to wild-type (ΔΔCt). (**B**) *P. aeruginosa* PAO1 wild-type was cultured either in DeMoss medium supplemented with low phosphate and high phosphate, respectively, or M9 minimal medium with 10 mM citrate or 10 mM glucose. The relative expressions were calculated from at least three biological and two technical replicas.

In order to further evaluate the activity of the two RRs on the promoter region of the co-regulated genes, we generated in addition to the *phoB* deletion mutant, also a *tctD* deletion and a double *phoB-tctD* deletion mutant in the PAO1 strain background. We then used RT-PCR to monitor gene expression of *glpQ, ugd* and *oprO* (harboring the PhoB/TctD motif) as well as of the TctD regulated gene *opdH* in the PAO1 wild-type and the respective mutants. The promoter of *ugd, glpQ* and *oprO* were clearly active in the PAO1 wild-type and their activity was reduced in the *phoB* and *phoB-tctD* double mutant (Mann–Whitney test *P* < 0.05) indicating that PhoB acts as a transcriptional activator (Figure 7A). These results corroborate the results of the RNA-seq analysis of the *phoB* mutant as compared to its PA14 wild-type (Table 1). Of note, RT-PCR in PAO1 revealed that also *ugd* which harbors a PhoB/TctD motif in its promoter region is positively regulated by PhoB (which was not found to be differentially regulated in the global RNA-seq analysis of the PA14 *phoB* mutant). In contrast, RT-PCR analysis revealed that gene expression of *ugd, glpQ* and *oprO* was enhanced in the *tctD* mutant. As opposed to this, the promoter activity of the *opdH* gene was reduced in the *tctD* and the *phoB-tctD* double mutant (Mann–Whintey test *P* < 0.05). These results indicate that TctD counteracts the activity of PhoB on the promoter region of the co-regulated genes.

To extend the picture, the PA14 wild-type was also cultivated under conditions, which either favored or inhibited *phoB* expression (DeMoss medium supplemented with low phosphate and high phosphate concentrations, respectively) or *tctD* expression (M9 minimal medium with 10 mM citrate or 10 mM glucose) (Figure 7B). The *tctD* or *tctD/phoB* mutants were not able to grow with citrate as sole carbon source. As expected, the three co-regulated genes *glpQ, oprO* and *ugd* exhibited increased expression under low phosphate conditions. Furthermore as reported before, we observed a strong induction of *opdH* expression if bacteria were grown in citrate (38), corroborating the finding that TctD activates *opdH* expression.

## DISCUSSION

Protein–protein interactions are important for the majority of biological functions and thus are subject of intensive studies (40). In recent years the extreme sensitivity of the *Gaussia princeps* luciferase GLuc has been exploited for the development of PCA to study the dynamics of protein–protein interactions in mammalian cells *in vitro* and *in vivo* (41,42). Furthermore, this system has been used to study protein–protein interactions in live bacterial cells (20). Beside its small size, GLuc possesses several advantageous characteristics: it is very stable over a wide pH range and in the presence of reactive compounds, its substrate can permeate cell membranes and GLuc does not require any cofactors to be active (43).

In this study, we demonstrated that the substrate coelenterazine which is oxidized by GLuc clearly penetrates the cell membranes of *P. aeruginosa* although we observed striking differences between the two *P. aeruginosa* strains PAO1 and PA14. The luminescence signals in a GLuc expressing PAO1 strain exceeded those of the GLuc expressing PA14 strains. Remarkably, this seemed to be due to either a lower penetration of the substrate through the PA14 membrane or due to an increased active export of the substrate from the cytoplasm, since the luminescence signal intensities did not differ between the two strains when the cells were lysed.

The split-GLuc PCA was successfully applied to systematically search for functional interactions of a diverse array of RRs of the PhoB/OmpR subfamily with PhoB in *P. aeruginosa*. The assay revealed a positive luminescence signal if split PhoB-CGluc and TctD-NGluc were introduced into PAO1, indicating a functional interaction of the two RRs *in vivo*. By the use of transcriptional profiling, ChIP-seq and a global motif scan we uncovered the regulons of the two RRs. The regulon of TctD is rather small and comprised overall 10 genes/operons including the *tctD* gene itself and the o*pdHtctCBA* operon. The respective promoter regions of the latter harbored a singular TctD binding motif. However, for seven of the remaining eight promoter regions we identified strong TctD and PhoB motifs. Interestingly in six of those promoters this was a quadripartite binding motif of PhoB/TctD while in one promoter region both a TctD and a PhoB motif was present, albeit with a larger spacing between the two motifs. It is reasonable to assume that a simultaneous binding of the two RRs to those promoter regions induced a positive luminescence signal of the split luciferase, albeit it cannot be excluded that the two RRs also functionally interact and associate before binding to their respective bipartite DNA binding sites. However, the regulons of PhoB and TctD are very different in size. Whereas we identified only 10 TctD binding sites, the primary PhoB regulon—as determined here by applying a combinatorial approach using ChIP-seq, RNA-seq and a motif scan—comprises as many as 188 direct PhoB binging sites throughout the chromosome. The activity of the large majority of those PhoB binding promoter regions was not affected by the absence or presence of TctD, indicating that functional interaction of PhoB and TctD may apply selectively at the promoter region of the TctD/PhoB co-regulated genes/operon structures.

Exemplary testing of three promoter regions of the co-regulated genes revealed that both RRs bind to the promoter regions as demonstrated by gel shift assays. However, the transcriptional regulators PhoB and TctD seem to act as opponents rather than exhibiting a cooperative activity. RNA-seq and RT-PCR analyses revealed that expression of selected genes was clearly dependent on PhoB, whereas presence of TctD repressed gene expression. Thus, it seems that binding of TctD directly down-stream of the PhoB DNA binding site represses transcription by inhibiting PhoB-dependent transcriptional activation of gene expression. Binding of TctD could repress transcription in two ways: either physically blocking the access of the RNA polymerase to the promoter or altering the topology of DNA in the vicinity of the promoter, thus impairing transcription initiation. Of note, all PhoB/TctD DNA binding sites were found within 73 and 227 base pairs upstream of the start codon and there was no strong −10/−35 motif, indicating that alternative sigma factors might be additionally involved in gene regulation.

PhoB/TctD cross-regulation at the level of binding of the RRs to adjacent DNA motifs on selected promoter regions seems to provide an effective way to integrate various environmental signals and to coordinate gene expression in the opportunistic pathogen *P. aeruginosa*. The two two-component RRs are phosphorylated by distinct histidine kinases that sense the availability of carbon sources and phosphate, respectively. The perception and the integration of those two important environmental signals is achieved by a simple titration of the relative amounts of the two phosphorylated RR exhibiting an activating versus an inhibitory activity, respectively. In conclusion, our results on two two-component signal transduction pathways in *P. aeruginosa* suggest that the bacteria might exploit cross-talk between them to adapt bacterial behavior to complex environments.

## DATA AVAILABILITY

All raw and processed data are available at the Gene Expression Omnibus (GEO) under the accession GSE64056.

## SUPPLEMENTARY DATA

Supplementary Data are available at NAR Online.

SUPPLEMENTARY DATA
