# Supplementary material for: Cross talk between the response regulators PhoB and TctD allows for the integration of diverse environmental signals in Pseudomonas aeruginosa
Source: Nucleic Acids Res. 2015 Jun 15;43(13):6413–25. doi: 10.1093/nar/gkv599 (PMC4513871; doi:10.1093/nar/gkv599)
Supplement: SUPPLEMENTARY DATA [file supp_43_13_6413__index.html]

Cross talk between the response regulators PhoB and TctD allows for the integration of diverse environmental signals in Pseudomonas aeruginosa — SUPPLEMENTARY DATA 

# Cross talk between the response regulators PhoB and TctD allows for the integration of diverse environmental signals in *Pseudomonas aeruginosa*

## SUPPLEMENTARY DATA

- SUPPLEMENTARY DATA
- SUPPLEMENTARY DATA
- SUPPLEMENTARY DATA
- SUPPLEMENTARY DATA
- SUPPLEMENTARY DATA
- SUPPLEMENTARY DATA
- SUPPLEMENTARY DATA
