# Supplementary material for: Cross talk between the response regulators PhoB and TctD allows for the integration of diverse environmental signals in Pseudomonas aeruginosa
Source: Nucleic Acids Res. 2015 Jun 15;43(13):6413–25. doi: 10.1093/nar/gkv599 (PMC4513871; doi:10.1093/nar/gkv599)
Supplement: SUPPLEMENTARY DATA [file supp_gkv599_nar-03615-v-2014-File009.docx]

**Supplementary Figure 1** Growth of PAO1 (OD_600_) (solid line) as well as the luminescence signal expressed as Relative Luminescence Units normalized to the bacterial OD_600_, RLU/OD_600_, was recorded over time under the addition of 0.1% L-arabinose. Mean RLU/OD_600_ values and standard deviation of three biological replicas are shown.

**Supplementary Table S1** Bacterial strains and plasmids used in this study.

**Supplementary Table S2** Primers used in this study.

**Supplementary Table S3** Differentially expressed genes in the *phoB* and *tctD* mutants as compared to the PA14 wild type.

**Supplementary Table S4** PhoB ChIP-seq enriched regions as detected by the MACS software.

**Supplementary Table S5** Genes defined to belong to the PhoB primary regulon.
