# Supplementary figures and images for: Cross talk between the response regulators PhoB and TctD allows for the integration of diverse environmental signals in Pseudomonas aeruginosa
Source: Nucleic Acids Res. 2015 Jun 15;43(13):6413–25. doi: 10.1093/nar/gkv599 (PMC4513871; doi:10.1093/nar/gkv599)

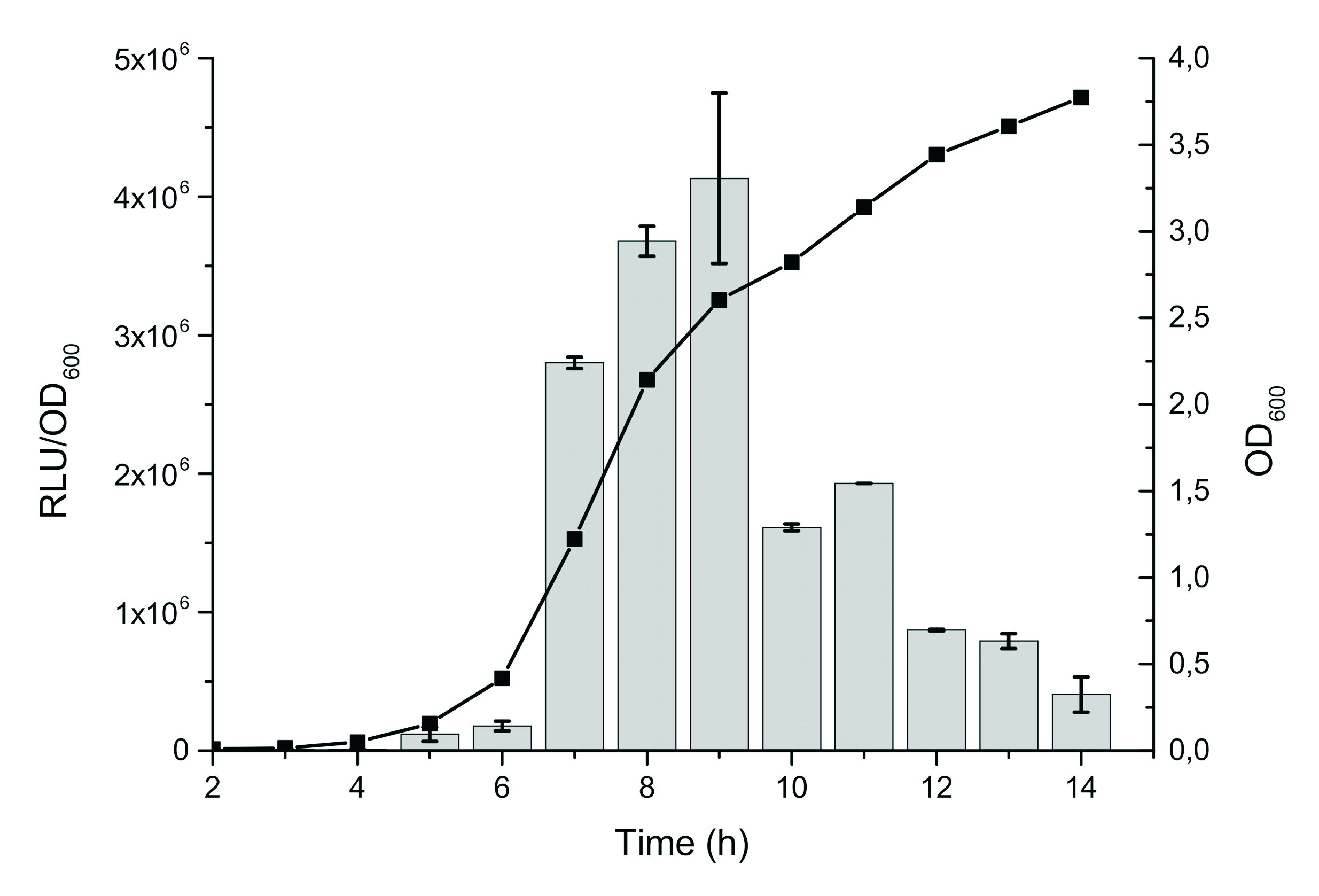

Supplement: SUPPLEMENTARY DATA [file supp_gkv599_nar-03615-v-2014-File010.tif]
