# Supplementary material for: Cross talk between the response regulators PhoB and TctD allows for the integration of diverse environmental signals in Pseudomonas aeruginosa
Source: Nucleic Acids Res. 2015 Jun 15;43(13):6413–25. doi: 10.1093/nar/gkv599 (PMC4513871; doi:10.1093/nar/gkv599)
Supplement: SUPPLEMENTARY DATA [file supp_gkv599_nar-03615-v-2014-File011.docx]

Table S1. Bacterial strains and plasmids used in this study

| *Escherichia coli*  DH5α | F^-^ endAl glnV44 thi-l recAl relAl gyrA96 deoR nupG φ8dlacZΔM15 Δ/lacZYA- argF)U169, hsdR17(rK- mK+), λ- | Woodcock et al., 1989 (50) |
| --- | --- | --- |
| *Escherichia coli*  S17-1 | C600::RP-4 2-(Tc::Mu) (Kn::Tn7) | Simon et al., 1983 |
| *Escherichia coli*  WM3064 | thi pro hsdR hsdM+recA ThrB1004 pro thi rpsL hsdS lacZΔM15 RP4-1360 Δ(araBAD)567 ΔdapA1341::[erm pir(wt)] | W. W. Metcalf, University of Illinois, Urbana |
| *Escherichia coli* BL21 (DE3) | F^-^ ompT hsdSB(rB^-^ mB^-^) gal dcm | Stratagene |
| PA14 | *Pseudomonas aeruginosa*  type strain | Liberati et al., 2006 (21) |
| PAO1 | *Pseudomonas aeruginosa*  type strain | Jacobs et al., 2003 (51) |
| PAO1 Δ*phoB* | Markerless deletion mutant of *phoB* | This study |
| PA14 Δ*phoB* | Markerless deletion mutant of *phoB* | This study |
| PA14 *tctD:tn* | RNA-seq was done with transposon mutant | Liberati et al., 2006 (21) |
| PAO1 *ΔtctD:FRT* | *tctD* deletion mutant | This study |
| PAO1 Δ*phoB*Δ*tctD*:*Gm* | *phoB tctD* double deletion mutant | This study |
| pUCP20 | Amp/Cb | West et al., 1994 |
| pHERD20T | Amp/Cb | Qiu et al., 2008 |
| pET21a(+) | Amp/Cb | Novagen |
| pBAD24-*gluc* | Plasmid bearing codon optimized gaussia luciferase encoding gene | R. Gerlach (35) |
| pHERD20T-*gluc* | Gluc gene cloned together with synthetic RBS site into EcoRI and XbaI | This study |
| pHERD20T*gluc*NNfus | Containing N and C-terminal Split-halves of *gluc* | This study |
| pHERD20T-P_BAD_-RR-N*gluc*-*phoB-*C*gluc* | Fusion of C*gluc* with *phoB* | This study |
| pHERD20T-P_BAD_-*phoB*-N*gluc*-*phoB-*C*gluc* | Fusion of C*gluc* with *phoB* and N*gluc* with *phoB* | This study |
| pHERD20T-P_BAD_-*bfmR*-N*gluc*-*phoB-*C*gluc* | Fusion of C*gluc* with *phoB* and N*gluc* with *bfmR* | This study |
| pHERD20T-P_BAD_-*creB*-N*gluc*-*phoB-*C*gluc* | Fusion of C*gluc* with *phoB* and N*gluc* with *creB* | This study |
| pHERD20T-P_BAD_-*gltR*-N*gluc*-*phoB-*C*gluc* | Fusion of C*gluc* with *phoB* and N*gluc* with *gltR* | This study |
| pHERD20T-P_BAD_-*amgR*-N*gluc*-*phoB-*C*glc* | Fusion of C*gluc* with *phoB* and N*gluc* with *amgR* | This study |
| pHERD20T-P_BAD_-*pmrA*-N*gluc*-*phoB-*C*gluc* | Fusion of C*gluc* with *phoB* and N*gluc* with *pmrA* | This study |
| pHERD20T-P_BAD_-*kdpE*-Ngluc-*phoB-*Cgluc | Fusion of C*gluc* with *phoB* and N*gluc* with *kdpE* | This study |
| pHERD20T-P_BAD_-*PA1437*-N*gluc*-*phoB-*C*gluc* | Fusion of C*gluc* with *phoB* and N*gluc* with *PA1437* | This study |
| pHERD20T-P_BAD_-*parR*-N*gluc*-*phoB-*C*gluc* | Fusion of C*gluc* with *phoB* and N*gluc* with *parR* | This study |
| pHERD20T-P_BAD_-*copR*-N*gluc*-*phoB-*C*gluc* | Fusion of C*gluc* with *phoB* and N*gluc* with *copR* | This study |
| pHERD20T-P_BAD_-*creB*-N*gluc*-*phoB-*C*gluc* | Fusion of C*gluc* with *phoB* and N*gluc* with *creB* | This study |
| pHERD20T-P_BAD_-*PA4381*-N*gluc*-*phoB-*C*gluc* | Fusion of C*gluc* with *phoB* and N*gluc* with *PA4381* | This study |
| pHERD20T-P_BAD_-*PA2479*-N*gluc*-*phoB-*C*gluc* | Fusion of C*gluc* with *phoB* and N*gluc* with *PA2479* | This study |
| pHERD20T-P_BAD_-*PA0929*-N*gluc*-*phoB-*C*gluc* | Fusion of C*gluc* with *phoB* and N*gluc* with *PA0929* | This study |
| pHERD20T-P_BAD_-*PA3077-*N*gluc*-*phoB-*C*gluc* | Fusion of C*gluc* with *phoB* and N*gluc* with *PA3077* | This study |
| pHERD20T-P_BAD_-*PA 2523*-N*gluc*-*phoB-*C*gluc* | Fusion of C*gluc* with *phoB* and N*gluc* with *PA2523* | This study |
| pHERD20T-P_BAD_-*PA 0765*-N*gluc*-*phoB-*C*gluc* | Fusion of C*gluc* with *phoB* and N*gluc* with *PA0756* | This study |
| pHERD20T-P_BAD_-*pfeR*-N*gluc*-*phoB-*C*gluc* | Fusion of C*gluc* with *phoB* and N*gluc* with *pfeR* | This study |
| pHERD20T-P_BAD_-*pilH*-N*gluc*-*phoB-*C*gluc* | Fusion of C*gluc* with *phoB* and N*gluc* with *pilH* | This study |
| pET21::phoB | *phoB* gene without stop codon cloned into NdeI-HindIII in MCS | This study |
| pET21::tctD | *tctD* gene without stop codon cloned into NdeI-XhoI in MCS | This study |
| \| pJN105 \|  \| 12 \| \| --- \| --- \| --- \| | Broad-host-range low-copy-number vector pBBR1-MCS5 harboring *araC*-P*_BAD_* cassette from pBAD18, Gm^r^ |  |
| pJN105-RBS-sigX-his8 | phoB ORF with optimized start and stop codon, preceding RBS and C-terminal 8×His coding sequence cloned into pJN105 using EcoRI and XbaI sites, Gm^r^ | This study |
| pUCP20 rev BamHI-*gluc*-EcoRI | *gluc* gene cloned in reverse order to lacZ promoter | This study |
| pPS586 | Gmr cassette flanked by FRT sites, Apr, Gmr | Hoang et al., 1998 |
| pEX18Ap | Plasmid for gene replacement; oriT+ sacB+, Ap^r^ | Hoang et al., 1998 |
