# Supplementary material for: Cross talk between the response regulators PhoB and TctD allows for the integration of diverse environmental signals in Pseudomonas aeruginosa
Source: Nucleic Acids Res. 2015 Jun 15;43(13):6413–25. doi: 10.1093/nar/gkv599 (PMC4513871; doi:10.1093/nar/gkv599)
Supplement: SUPPLEMENTARY DATA [file supp_gkv599_nar-03615-v-2014-File012.docx]

Table S2. Primers used in the study

| GlucEcoRIRBS | CGGAATTCCaaagaggagaaatactagatgaaaccgaccgaaaacaacga |
| --- | --- |
| GlucXbaIStop | GCTCTAGAGCttattaatcgccacccgcacc |
| phoB rbs KpnI F | GGggtaccaagaggagaaatactagATGGTTGGCAAGACAATCCTCATC |
| phoB linker XbaI R | GCTCTAGAGCTCTTGGTGGAGAAACGATAGC |
| phoB F | GgaattcCaaagaggagaaatactagATGGTTGGCAAGACAATCCTCATC |
| phoB R | GCgagctcGCTCTTGGTGGAGAAACGATAGC |
| PA4101F | cggaaTTCCaaagaggagaaatactagATGGAGCATGTCGATCACATCCTGA |
| PA4101R | CCgagctcTGGATGGGCCTCGACCAGTCG |
| PA0463F | cggaaTTCCaaagaggagaaatactagATGCCGCATATCCTGATCGTCGAAGAT |
| PA0463R | CCgagctcGGCGTGGTCCGGGCTGTAGC |
| PA3192F | cggaaTTCCaaagaggagaaatactagGTGAGCGCGAACGGACGATCGA |
| PA3192R | CCgagctcTGGCTGCAGGTGCGGCCGCA |
| PA5200F | cggaaTTCCaaagaggagaaatactagATGTCGAACCCTGCCGCCCTG |
| PA5200R | CcgagctcGGCCTTGCGCGCGTTGCCGT |
| PA4776F | cggaaTTCCaaagaggagaaatactagATGAGAATACTGCTGGCCGAGGACG |
| PA4776R | CCgagctcGGGCGCCGGCTGGTCGATGC |
| PA1637F | cggaaTTCCaaagaggagaaatactagATGACCCAATTGCAGAACAGCATCCT |
| PA1637R | CCgagctcGCTGTCGCGCAGGCGATAACC |
| PA1437F | cggaaTTCCaaagaggagaaatactagATGCGGGTACTGATTGTCGAGGAC |
| PA1437R | Ccgagctc GCCCGACTCCGCCAGCGCAC |
| PA1799F | cggaaTTCCaaagaggagaaatactagATGGACTGCCCTACCCTCAGCAAG |
| PA1799R | CCGAGCTCCCAGCCCAGCGGGT |
| PA2809F | cggaaTTCCaaagaggagaaatactagATGAAACTGCTGATCGTCGAAGACGA |
| PA2809R | CCgagctcTTCGTCGCGCTCTTCGAGAACGT |
| PA4381 F | cggaaTTCCaaagaggagaaatactagATGCGAATACTGGTGGTCGAAGAC |
| PA4381R | CCgagctcTACTCCATTCGGCTCCTCCGC |
| PA2479F | cggaaTTCCaaagaggagaaatactagATGCATGTACTGCTCACCGAAGAC |
| PA2479R | CCgagctcTGCGTCGTCTCCGTCGCCGC |
| PA0929F | cggaaTTCCaaagaggagaaatactagATGTTCCCATCCCTGACGCCCGA |
| PA0929R | CCgagctcTGGCGCCGGTGTCAGGACGT |
| PA3077F | cggaaTTCCaaagaggagaaatactagATGCATATCCACGTACTCGTCGTCGA |
| PA3077R | CCgagctcTTTGCGCTCCAGCTGGAAGCCG |
| PA2523F | cggaaTTCCaaagaggagaaatactagATGCGCATCCTTATTATCGAAGATGAAG |
| PA2523R | CCgagctcTCGGCGCGCTTCCAGGACGT |
| PA0756F | cggaaTTCCaaagaggagaaatactagATGCGCATCCTTCTGGTGGAAGAT |
| PA0756R | CCgagctcGTCACCCTGCGCCTCCAGCA |
| PA2686F | cggaaTTCCaaagaggagaaatactagATGATCGTACAACCCAGTGCCGTC |
| PA2686R | CCgagctcGGGGTGTTCCGTCTCCACCAG |
| PA0409F | cggaaTTCCaaagaggagaaatactagATGGCTCGTATTTTGATTGTTGATGACTC |
| PA0409R | CCgagctcGCCCGCCAGCACCGCATTGA |
| ΔphoB 1 | CGAATTCTGTCCTGGATGAAGGTATAAAGG |
| ΔphoB overlap rev | ACGATAGCCCTTGCCAACCATGGTCTTG |
| ΔphoB overlap fw | GTTGGCAAGGGCTATCGTTTCTCCACCAA |
| ΔphoB 2 | TCCTGGATCCGGTCGATC |
| Δ*tctD* UP F EcoRI | CGGAATTC ACGGTGCCCTGGGTGTAGC |
| Δ*tctD*  UP R | TGGCGGATCCGCGGATGATCTTCCACCAGAAG |
| Δ*tctD*  DW F | TCCGCGGATCCGCCATCGAGATCTACGTCCAC |
| Δ*tctD* DW R XbaI | GCTCTAGA GCTGCAGCAGGCTCACCAC |
| PA14_04550 F | CCCAAGCTT CAACGACAGGCAGGGAAACG |
| PA14_04550 R | CGCGGATCC GACGGGTCCTCGTCGGCG |
| PA14_21610 F | CCCAAGCTT ACCCATATCGAATAACCCGA |
| PA14_21610 R | CGCGGATCC CGAAGATTTCCCCTTAATGG |
| PA14_38360 F | CCCAAGCTT CGATGCCGGCTGCCTTCGATCT |
| PA14_38360 R | CGCGGATCC GGCGTCGTCCTCCGCTGGCC |
| PA14_56560 F | CCCAAGCTT GCATCCTGCCGAGGGTGCCA |
| PA14_56560 R | CGCGGATCC GCCTGGGCCTCCTTGGGCCG |
| PA14_20491 F | CCCAAGCTT ACTGGCCCAGGAGAAGCGAT |
| PA14_20491 R | CGCGGATCC GCTGCACCTCCAGGGTTGCG |
| PhoB F NdeI | GATCCATATGATGGTTGGCAAGACAATC |
| PhoB HindIII | GATCAAGCTTGCTCTTGGTGGAGAAACG |
| TctD F NdeI | GGAATTCCATATGATGCGCATCCTTCTGGTG |
| TctD R XhoI | CCGCTCGAGGTCACCCTGCGCCTCC |
| glpQ-RT F | **AGGGTGTGTTCCGGTACGTA** |
| glpQ-RT R | **GACACCGGCAACGTACAAC** |
| orpO-RT F | **GGTGTTGCCATTCTTGGTATAGA** |
| orpO-RT R | **GATCAAGACCAAGGGTGGC** |
| ugd-RT F | **TATGTGGGACTGGTGACGG** |
| ugd-RT R | **GCAGGATGCTTTCCAGGC** |
| opdH-RT F | **TCCTTGATCTCCTTCGAGGT** |
| opdH-RT R | **AAGGTCTCGCAGAGCGA** |
| rspL-RT F | GACAAGAGCGACGTGCCT |
| rspL -RT R | ACGGATCAGCACTACGCTGT |
